# Epigenetic changes in the estrogen receptor α gene promoter: implications in sociosexual behaviors

**DOI:** 10.3389/fnins.2014.00344

**Published:** 2014-10-28

**Authors:** Ken Ichi Matsuda

**Affiliations:** Department of Anatomy and Neurobiology, Graduate School of Medical Science, Kyoto Prefectural University of MedicineKyoto, Japan

**Keywords:** estrogen receptor α, epigenetics, histone acetylation, histone deacetylation, DNA methylation, sexual differentiation, sexual behavior, sociosexual behavior

## Abstract

Estrogen action through estrogen receptor α (ERα) is involved in the control of sexual and social behaviors in adult mammals. Alteration of ERα gene activity mediated by epigenetic mechanisms, such as histone modifications and DNA methylation, in particular brain areas appears to be crucial for determining the extents of these behaviors between the sexes and among individuals within the same sex. This review provides a summary of the epigenetic changes in the ERα gene promoter that correlate with sociosexual behaviors.

## ERα and its gene promoter

Estrogen receptor α (ERα) is a member of the nuclear receptor superfamily of ligand-dependent transcription factors that regulate expression of target genes (Evans, 1988; Kawata, 1995; Parker, 1995; Matsuda et al., 2002; McCarthy, 2008). ERα has a typical nuclear receptor structure with at least three functional domains: the ligand-binding domain located in the C-terminal half of the protein, the DNA-binding domain located centrally, and a variable transactivation domain located in the N-terminal region. Upon activation by a ligand, estradiol, ERα forms a homodimer within the nucleus and the dimer complex binds to specific regulatory DNA sequences, which are referred to as estrogen responsive elements (EREs), in promoter or enhancer regions of target genes. After binding to an ERE, the ERα dimer recruits transcription co-factors, which leads to gene activation and transcription. Following transcription, mRNA is translated into proteins that are the ultimate outcome of the hormone responses. Alternatively, accumulating evidence suggests that rapid non-genomic actions of ERα initiated at the plasma membrane through induction of protein phosphorylation-mediated signal transduction pathways are also crucial in estrogenic responses (Vasudevan and Pfaff, 2008; Sakamoto et al., 2012). These characteristics of ERα are common to the other estrogen receptor subtype, ERβ (Koehler et al., 2005).

Expression of the ERα gene is controlled by multiple promoters located upstream of the first coding exon (Kos et al., 2001; Wilson et al., 2008). In rats, at least four different promoters (C, 0S, 0N, and 0B) that can initiate transcription have been identified and shown to be utilized in an organ- and tissue-specific manner. The ERα gene transcript from the 0B promoter (also designated as the 1B promoter; Freyschuss and Grandien, 1996; Champagne et al., 2006), which corresponds to the C promoter in humans and mice, is expressed in brain areas involved in sociosexual behaviors, such as the bed nucleus of the stria terminalis (BNST) (Emery and Sachs, 1976) (Numan, 1996; Numan and Woodside, 2010), the medial preoptic area (MPOA) (Larsson and Heimer, 1964), and hypothalamic and amygdaloid nuclei (Kawata, 1995; McCarthy, 2008), as well as in the anterior pituitary, ovary and uterus (Kato et al., 1998).

## ERα in sociosexual behaviors

Gene targeting in mice has shown that ERα contributes to various brain functions, including regulation of sociosexual behaviors in both sexes (Rissman et al., 1997; Tetel and Pfaff, 2010).

### Female sexual behavior

ERα knockout (ERαKO) female mice, in which the ERα gene is disrupted in both alleles throughout the body, completely lack lordosis behavior, a typical female sexual behavior (Ogawa et al., 1996). ERαKO females are also deficient in sexual interactions that precede the lordosis response (Ogawa et al., 1998a). The estradiol level in gonadally intact ERαKO females is elevated compared to that in wild type females, and thus expression of ERα in the brain is critical for induction of female sexual behavior. However, these studies in ERαKO mice did not clarify whether the deficits were caused by a lack of ERα activation during development or in adulthood.

Spatiotemporal knockdown of ERα mRNA (ERαKD) mediated by infection with adeno-associated virus (AAV) expressing small hairpin (sh) RNA against ERα mRNA has been conducted in adult female mice. When gene silencing was restricted to the bilateral ventromedial nucleus of hypothalamus (VMH), where ERα is strongly expressed, the mice exhibited no sexual behavior (Musatov et al., 2006), indicating that ERα function in the VMH in adulthood is a key regulator of female sexual behavior. Female mice in which expression of ERα was silenced in the MPOA also exhibited significant reduction in receptive and rejective female sexual behaviors (Ribeiro et al., 2012).

### Male sexual behavior

In males, estrogen is produced from testosterone by the enzymatic activity of aromatase in the brain and is known to regulate sexual behavior. Male ERαKO mice show significant impairment in some components of sexual behavior compared with wild type mice. ERαKO mice exhibit a normal motivation to mount females, but reduced levels of intromission and no ejaculation (Ogawa et al., 1997; Wersinger et al., 1997; Ogawa et al., 1998b; Scordalakes and Rissman, 2003).

Brain regions responsible for ERα-mediated regulation of male sexual behavior have been examined using ERαKD by AAV infection. Male sexual behavior was greatly reduced when ERα expression was silenced in the MPOA (Sano et al., 2013). In MPOA ERαKD mice, mount motivation and intromission were reduced, suggesting that ERα expressed in the MPOA in adulthood is involved in the control of male sexual motivation and behavior. Silencing ERα expression in the VMH also caused a reduction in male sexual behavior, particularly in the number of intromissions (Sano et al., 2013). This result indicates that ERα function in the VMH is also important for the expression of male sexual behavior.

### Female social behavior

ERαKO females show increased aggression toward other females (Ogawa et al., 1996). Gonadally intact ERαKO females vigorously attack gonadectomized and steroid-primed female intruder mice. Gonadectomized and steroid-primed ERαKO females placed in the home cage of males that showed sexual behavior to wild type females showed extreme rejection of male mounts, whereas gonadally intact ERαKO females were vigorously attacked by the males (Ogawa et al., 1996, 1998a). Similarly, ERα gene silencing in the VMH caused steroid-primed females to reject males (Spiteri et al., 2010a,b). In contrast, ERαKD in the MPOA decreased aggression toward male intruders, as well as social investigation behaviors consisting of genital sniffing, touching the back and chasing (Spiteri et al., 2012).

ERα signaling also contributes to the induction of maternal behavior toward newborn pups. ERαKO females exhibited greatly reduced pup retrieval behavior compared with wild type controls (Ogawa et al., 1996). Silencing of ERα mRNA in the MPOA almost completely abolished maternal behaviors, including nursing and licking the pups, and significantly increased latency to pup retrieval (Ribeiro et al., 2012).

### Male social behavior

Estradiol contributes to male aggressive behaviors at least partially via ERα. Male-typical offensive attacks are rarely observed in gonadally intact or gonadectomized and androgen-replaced ERαKO males (Ogawa et al., 1997, 1998b). ERαKD in the VMH reduces aggressive behavior, but this effect is not seen for ERαKD in the MPOA (Sano et al., 2013).

## Epigenetic changes in the ERα gene promoter

Studies using ERα gene targeting techniques suggest that alteration of sensitivity to estrogen by changing the expression level of ERα in specific brain regions is a crucial feature in the control of sociosexual behaviors.

### Sex difference

The sex of the brain is mostly determined by the effects of androgen and its metabolite, estradiol. In rodents, androgen is transiently secreted from the testes during a critical perinatal period, the so-called androgen surge, and organizes the developing brain into a masculinized phenotype (Arnold and Gorski, 1984; Kawata, 1995; Matsuda et al., 2008; McCarthy, 2008). Androgen does not affect the brain directly; instead masculinization is largely mediated by estradiol converted from testosterone by aromatase in the brain. The presence or absence of brief exposure to estradiol during the perinatal period creates permanent sex differences in the brain including lasting sex differences in the expression of several genes. ERα expression in the preoptic area (POA) is higher in females than in males from postnatal day 2 through adulthood (DonCarlos and Handa, 1994; DonCarlos, 1996; Yokosuka et al., 1997). Thus, how the early effects of estrogen on the developing brain are permanently maintained is a fundamental issue in the study of sexual differentiation of the brain. Epigenetic mechanisms are emerging as important mediators for the maintenance of the hormonal effects (Keverne and Curley, 2008; McCarthy and Crews, 2008; Matsuda et al., 2012).

**Figure 1:**
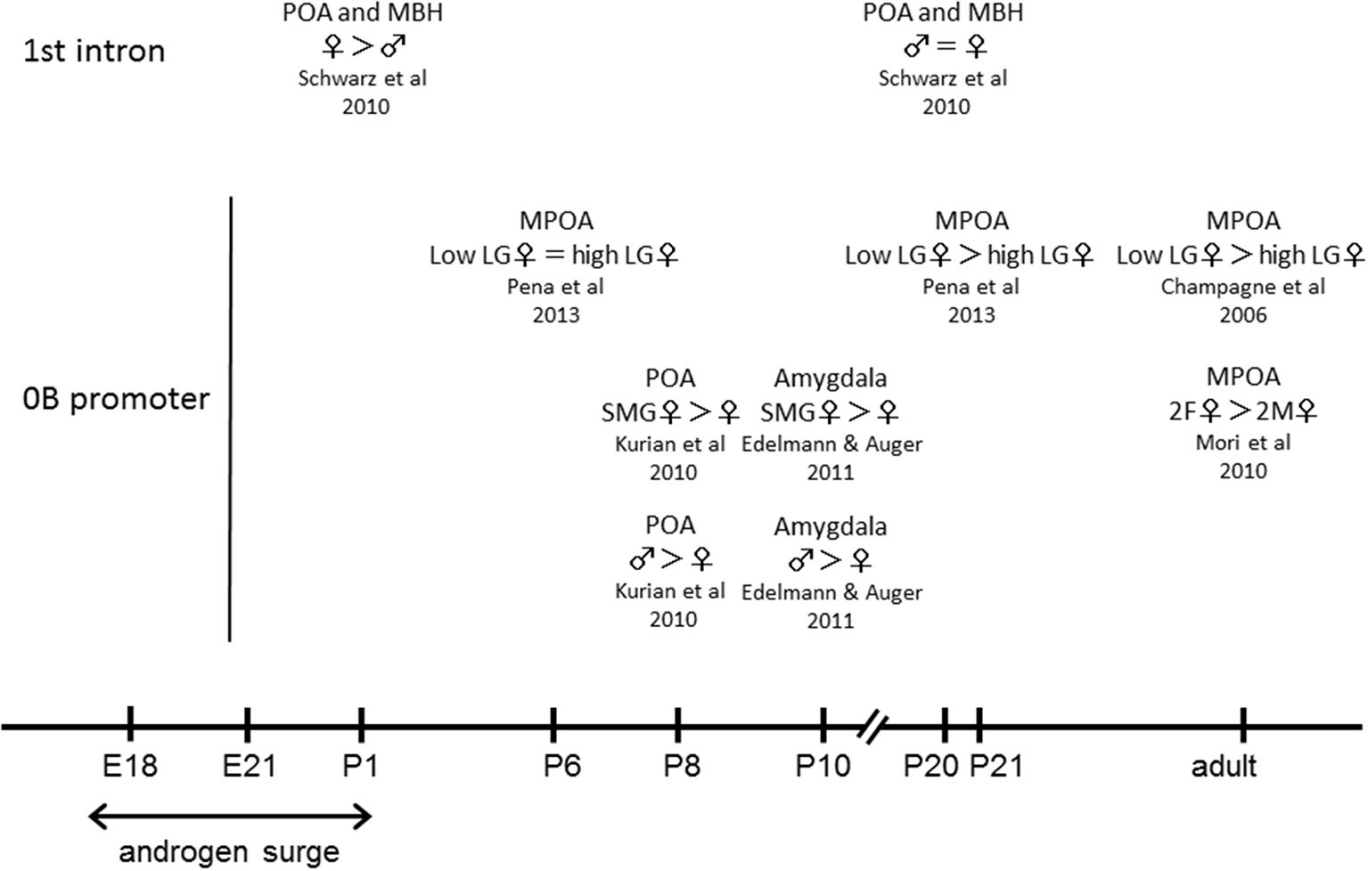
**Summary of DNA methylation status in the ERα gene in association with sociosexual behaviors**. E, embryonic day; P, postnatal day.

DNA methylation is a well characterized epigenetic change that contributes widely to transcriptional regulation (Nakao, 2001; Felsenfeld and Groudine, 2003). In the genome, the 5 position of the cytosine pyrimidine ring in the 5′-CpG-3′ dinucleotide is frequently modified with a methyl group. In general, the extent of CpG methylation in a promoter region is inversely correlated with the transcription level of the gene: higher methylation causes suppressed gene expression. The DNA methylation status of the CpG-rich region in the 1st intron of the ERα gene across the life span has been examined in the POA and the mediobasal hypothalamus (MBH), which includes the VMH (Schwarz et al., 2010). On postnatal day 1, during the critical period of sexual differentiation, two of seven CpG sites (one of these sites differs between the POA and MBH) have a significantly lower methylation rate in males than in females in both the POA and MBH (Figure 1). This difference is a result of estradiol exposure because treatment of females with estradiol 24 h before sample collection induces a methylation pattern identical to that in males. These site specific modifications of DNA methylation may be involved in the maintenance of ERα expression in males to facilitate the effect of estradiol during the androgen surge.

The histone acetylation status in the ERα gene promoter also shows a sex difference during the critical perinatal period. Histone acetylation is a well-characterized epigenetic modification that is important in transcriptional regulation (Kouzarides, 2007; Graff and Tsai, 2013). Histone acetylation neutralizes the positive charge of the histone tail and reduces its attraction to the negatively charged DNA, thereby loosening the nucleosome and allowing access of transcriptional factors, thus enhancing gene transcription. Acetylation levels of histone H4 at the ERα 0B promoter in the MPOA are higher in males than females on embryonic day 21 (Matsuda et al., 2011), suggesting prevention of downregulation of ERα expression in males.

The extent of methylation of CpG sites in the 1st intron of the ERα gene increases through development in both male and female MPOA and the sex difference detected on postnatal day 1 is abolished by postnatal day 20 (Schwarz et al., 2010) (Figure 1). In addition, an analysis of DNA methylation of the ERα 0B promoter in the POA on postnatal day 8 showed that the average methylation across 17 CpG sites was significantly higher in males compared with females (Kurian et al., 2010) (Figure 1). Two of the 17 CpG sites had significantly greater methylation in males and methylation at 6 other CpG sites was detected only in males. Estradiol treatment of females in the neonatal period increased methylation of the ERα promoter to a similar level to that in males. These findings suggest that sex differences in ERα gene expression may result from sex differences in DNA methylation patterns. A similar difference of methylation pattern at a specific CpG site in the ERα promoter has been seen in the amygdala (Edelmann and Auger, 2011), a brain area important for social and emotional processing, on postnatal day 10 (Figure 1).

**Figure 2:**
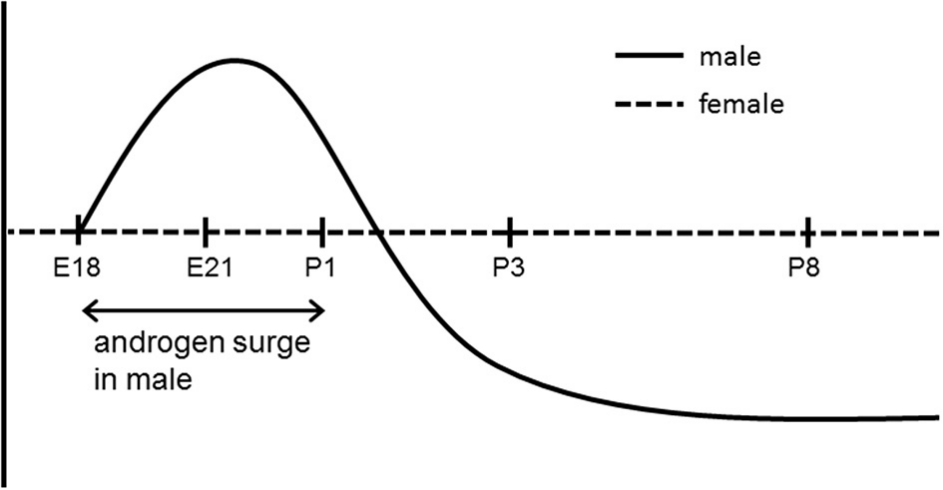
**Developmental changes in histone acetylation status in the ERα 0B promoter in the preoptic area in male and female rat**. E, embryonic day; P, postnatal day.

The histone acetylation status is inversely correlated with DNA methylation (Matsuda et al., 2011). Histone H4 acetylation differences in the ERα 0B promoter on embryonic day 21 were rearranged by postnatal day 3, at which time acetylation levels in males declined in correspondence with the developmental decrease in testosterone. The acetylation status of histones is controlled by the balance of enzymatic activity of histone acetyltransferases and histone deacetylases (HDACs), which remove the acetyl group from an acetylated histone. Thus, HDAC activity during the early postnatal period may be involved in the regulation of sexually dimorphic ERα expression in the MPOA (Figure 2). HDAC2 and -4, which are expressed in the developing brain and are related to steroid hormone signaling (Leong et al., 2005; Bicaku et al., 2008; Graff and Tsai, 2013), have been identified as candidate molecules regulating this process (Matsuda et al., 2011). The amount of HDAC2 and -4 binding to the ERα promoter on postnatal day 1 is higher in males than in females in the MPOA, while mRNA levels for HDAC2 and −4 do not differ between the sexes. The importance of HDAC activity in masculinization of the brain in the early postnatal period has been shown by both behavioral and morphological analyses. Inhibition of HDACs *in vivo* by intracerebroventricular infusion of a HDAC inhibitor (trichostatin A) or an antisense oligodeoxynucleotide directed against mRNA for HDAC2 and −4 in newborn male rats results in significant reduction of male sexual behavior in adulthood (Matsuda et al., 2011). Administration of another HDAC inhibitor (valproic acid) to male mice on postnatal days 1 and 2 eliminates the development of the sex difference in the volume of the principal nucleus of the BNST (Murray et al., 2009), which is normally larger in males than females.

**Figure 3:**
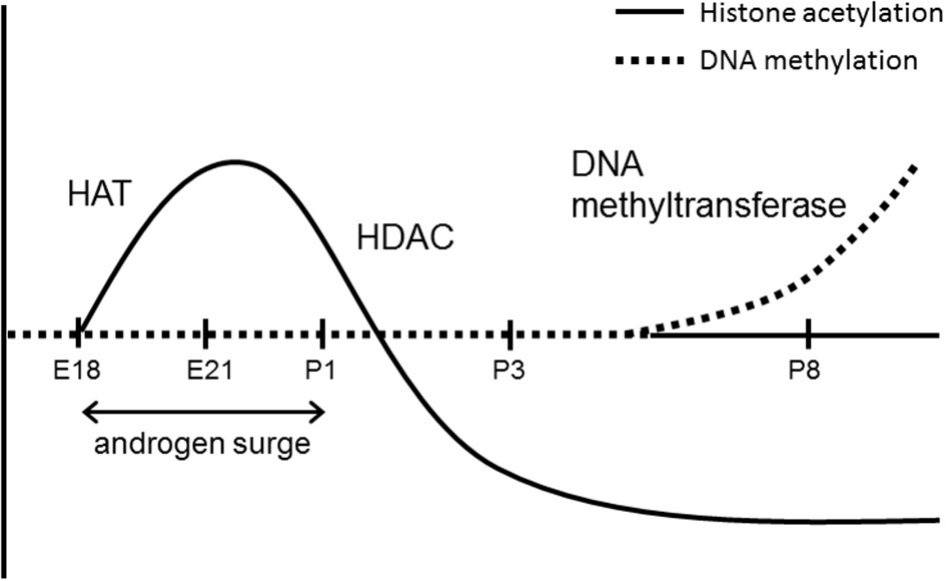
**Developmental changes in epigenetic status in the ER α 0B promoter in the preoptic area in male rat**. E, embryonic day; P, postnatal day.

These findings provide insights into the molecular mechanisms underlying the developmental consequences of sexually dimorphic ERα expression mediated by epigenetic modifications in the MPOA. During the prenatal androgen surge and subsequent activation of ERα, the acetylation status of histones in the ERα promoter region is increased in males to maintain ERα expression. After the androgen surge, inactivation of ERα due to the decline in ligand levels leads to recruitment of HDAC2 and -4 to promoters and the acetylation status of the promoter is reduced (Figure 2). Following the change in histone acetylation, methylation of DNA in the ERα promoter region is increased to a greater extent in males (Figure 3). This results in continuous lower expression of ERα compared with females, which is appropriate for execution of masculinized brain functions. These processes are not evident in females due to the absence of an androgen surge, and the consequent higher sensitivity to estrogen with higher expression of ERα may induce feminized brain functions.

### Individual differences

As described above, perinatal estradiol exposure contributes to lasting sex differences in ERα expression. However, early social experience can also alter ERα expression and associated behaviors. Variations in maternal care in rats distinguished by levels of whole-body licking and grooming (LG) by the dam exert a lasting influence on some neuroendocrine and behavioral characteristics of offspring in adulthood (Francis et al., 1999; Liu et al., 2000; Champagne et al., 2001; Cameron et al., 2005, 2008a,b; Prior et al., 2013). Offspring of dams that display high levels of LG (high LG) exhibit more modest hypothalamic-pituitary-adrenal responses to stress, enhanced cognitive ability, a higher level of maternal behavior, and altered sexual behavior in comparison to offspring of dams with low levels of LG (low LG). The effect of an individual difference in maternal behavior is transmitted across generations (Champagne and Meaney, 2007). Adult female offspring of high LG mothers display increased pup LG, compared with adult female offspring of low LG mothers. Cross-fostering, in which pups born to high-LG mothers are fostered at birth to low-LG mothers and vice versa, has shown a direct relationship between maternal care actually received and individual characteristics, suggesting that an epigenetic mechanism underlies the transgenerational inheritance of the individual behavioral differences. Variation of neonatal maternal care has been associated with ERα expression (Champagne et al., 2003) and DNA methylation of the ERα promoter in the MPOA (Champagne et al., 2006). Females that received high LG exhibited elevated ERα expression in adulthood compared with females that received low LG. DNA methylation patterns across the ERα 0B promoter differed significantly, with 7 of 14 CpG sites exhibiting significantly greater methylation in offspring of low LG dams compared to those from high LG dams (Figure 1). These findings suggest that environmental differences during development are programmed in the brain as a different pattern of epigenetic marks, and that this leads to differences in neuroendocrine and behavioral characteristics after maturity.

Examination of the developmental emergence of LG-mediated epigenetic variation (Pena et al., 2013) indicated a significant difference in DNA methylation rate at the ERα 0b promoter between high LG and low LG individuals on postnatal day 21, but not on postnatal day 6 (Figure 1), concomitant with the appearance of different ERα mRNA expression. Another epigenetic change, histone methylation, which is catalyzed by histone methyltransferases (HMT), does not change the overall charge of the histone tail, but increases basicity and hydrophobicity, which enhances histone affinity for DNA (Zhang and Reinberg, 2001; Martin and Zhang, 2005). Therefore, histone methylation is generally correlated with transcriptional repression, although methylation of some residues can result in transcriptional activation. Histone H3 trimethylation at lysine 9 (H3K9me3) and lysine 4 (H3K4me3) are epigenetic marks for repressed and active gene transcription, respectively. Comparison of the histone methylation status at the ERα 0B promoter in the MPOA between high LG and low LG females showed reduced H3K9me3 and increased H3K4me3 in high LG offspring on postnatal day 21, but not on postnatal day 6. These findings suggest that the influence of the amount of maternal care on epigenetic effects is apparent between postnatal days 6 and 21.

There is a difference between the sexes in the amount of maternal care. Mother rats preferentially lick and groom their male offspring more than their female offspring (Moore, 1992). This phenomenon implies that somatosensory stimuli associated with maternal grooming, as well as hormone exposure, influence brain masculinization. Simulated maternal grooming (SMG) by stimulation of the anogenital region of female pups with a paintbrush from postnatal days 5 to 7 increases ERα 0B promoter CpG methylation to a similar level to that in males on postnatal day 8 (Kurian et al., 2010) (Figure 1). ERα expression in the POA on postnatal day 10 was significantly reduced in female pups that received SMG compared to control female pups. These results suggest that maternal grooming may contribute to brain sex organization through programming differences in ERα expression through the epigenetic machinery. A similar effect of SMG on the methylation pattern at a specific CpG site in the ERα promoter has been detected in the amygdala on postnatal day 10 (Edelmann and Auger, 2011) (Figure 1). However, there is a difference in the direction of the effect of maternal care between the two studies: ERα expression was enhanced in high LG females, but reduced in SMG-stimulated females. This may indicate that SMG does not exhibit actual maternal grooming effect, but sensory stimulation during neonatal period has lasting effect on the expression of ERα gene in the POA by altering DNA methylation status of its promoter.

In addition to the postnatal social, physiological and environmental stimuli, differences in the embryonic hormonal milieu can also have a lasting influence on the development of sociosexual behavior in the offspring brain, resulting in individual variation of behavioral characteristics in adulthood within the same sex. In polytocous animals, the sex-specific positioning of fetuses can result in a natural variation of the hormonal environment during intrauterine development due to diffusion of androgen from neighboring male siblings. During the late gestational period, both the blood and brain concentrations of testosterone are higher in female fetuses that grow between two male siblings (2M females) compared with growth between two female siblings (2F females) (vom Saal and Bronson, 1980; Pei et al., 2006). Corresponding to this different level of androgen exposure, 2M females show greater aggressiveness and less sexual receptivity than 2F females in adulthood (vom Saal, 1984, 1989). It can be hypothesized that there may be intrauterine position-related differential ERα expression in the VMH, and ERα expression levels have been found to differ between 2M and 2F female offspring (Mori et al., 2010), with ERα expression in the VMH being higher in 2M females than 2F females. CpG sites across the ERα 0b promoter region in the VMH were more densely methylated in 2F females than in 2M females (Figure 1), showing a negative correlation between ERα expression levels in the VMH and DNA methylation frequency in the ERα promoter. These findings indicate that programming effects induced by the intrauterine position may be mediated by epigenetic modification.

## Conclusion

ERα expressed in specific brain areas controls various sociosexual behaviors in both sexes. The ERα level is correlated with differences in the magnitude of expression of these behaviors between the sexes and among individuals. Epigenetic programming appears to play central roles in the lasting regulation of ERα expression in response to the hormonal, social, and physiological environment during development. It will be of interest to determine the mechanisms that link these environmental cues to patterns of epigenetic modification in the ERα promoter.

### Conflict of interest statement

The author declares that the research was conducted in the absence of any commercial or financial relationships that could be construed as a potential conflict of interest.
